# Spatiotemporal consistency analysis of cerebral small vessel disease: an rs-fMRI study

**DOI:** 10.3389/fnins.2024.1385960

**Published:** 2024-05-22

**Authors:** Jie Yang, Rui Xiao, Yujian Liu, Chaoliang He, Limei Han, Xiaoya Xu, Meining Chen, Jianquan Zhong

**Affiliations:** ^1^Department of Radiology, Zigong First People’s Hospital, Zigong, China; ^2^Sichuan Vocational College of Health and Rehabilitation, Zigong, China; ^3^North Sichuan Medical College, Nanchong, China; ^4^Department of Neurology, Zigong First People’s Hospital, Zigong, China; ^5^MR Research and Collaboration, Siemens Healthineers, Shanghai, China

**Keywords:** cerebral small vessel disease, cognition impairment, four-dimensional (spatiotemporal) consistency of local neural activity, rs-fMRI, SVD score

## Abstract

**Introduction:**

Cerebral small vessel disease (SVD) affects older adults, but traditional approaches have limited the understanding of the neural mechanisms of SVD. This study aimed to explore the effects of SVD on brain regions and its association with cognitive decline using the four-dimensional (spatiotemporal) consistency of local neural activity (FOCA) method.

**Methods:**

Magnetic resonance imaging data from 42 patients with SVD and 38 healthy controls (HCs) were analyzed using the FOCA values. A two-sample *t* test was performed to compare the differences in FOCA values in the brain between the HCs and SVD groups. Pearson correlation analysis was conducted to analyze the association of various brain regions with SVD scores.

**Results:**

The results revealed that the FOCA values in the right frontal_inf_oper, right temporal_pole_sup, and default mode network decreased, whereas those in the temporal_inf, hippocampus, basal ganglia, and cerebellum increased, in patients with SVD. Most of these varying brain regions were negatively correlated with SVD scores.

**Discussion:**

This study suggested that the FOCA approach might have the potential to provide useful insights into the understanding of the neurophysiologic mechanisms of patients with SVD.

## 1 Introduction

Cerebral small vessel disease (SVD) is a prevalent, chronic, and progressive vascular disease among elderly people (Ter Telgte et al., 2018). It encompasses a variety of pathologies and etiologies affecting cerebral small arterioles, arterioles, venules, and capillaries (Cuadrado-Godia et al., 2018). Its main clinical manifestations include various neurologic disorders, such as stroke, cognitive decline, Alzheimer’s disease, and abnormal gait. Clinically, it has been found that SVD has common small vessel pathologies and blood-brain barrier (BBB) impairment, and hence, SVD is recognized as a “whole-brain disease” (Shi and Wardlaw, 2016; Li et al., 2018; Ter Telgte et al., 2018; Chojdak-Łukasiewicz et al., 2021). The pathologic mechanisms involved in SVD still remain largely elusive. Imaging markers play a crucial role in diagnosing SVD because of its *in vivo* invisibility. These markers include white matter hyperintensities (WMH), lacunar infarction (LI), subcortical infarction, cerebral microbleeds (CMBs), and cerebral atrophy (Pantoni, 2010; Litak et al., 2020). Previous studies have proposed a total SVD score based on imaging markers (Staals et al., 2014), which provides a more complete estimate of the full impact of SVD on the brain and correlates with cognitive ability (Staals et al., 2015).

Resting-state functional magnetic resonance imaging (rs-fMRI) is a powerful neuroimaging tool widely used to detect intrinsic brain activity during rest. Several previous studies used amplitude of low-frequency fluctuations (ALFF), regional homogeneity (ReHo), and functional connectivity density (FCD) methods to investigate the impact of SVD on various regions of the brain. They analyzed the changes in ALFF in the whole brain of patients with subcortical ischemic vascular dementia, revealing that the ALFF values in the precuneus, bilateral anterior cingulate cortex, insula, and hippocampus were higher than in the normal group (Liu et al., 2014). Moreover, Ding et al. explored the changes in brain functional connectivity in patients with white matter lesion (WMLs) using the FCD method. They noted the involvement of the regions with altered FCD in cortical regions and caudate (Ding et al., 2016). The ReHo method was used to investigate the changes in neural consistency in patients with WMLs. The results revealed that the decrease in ReHo mainly occurred in the precerebral region, whereas the increase in ReHo mainly occurred in the posterior region (Ding et al., 2017; Orsolini et al., 2021). However, all of the aforementioned measures focused only on the temporal correlation of local adjacent voxels (temporal consistency). They neglected to consider the effect of spatial consistency of spontaneous brain activity signals between neighbors. This might lead to missing useful information in understanding the neuropathologic mechanisms in patients with SVD.

The four-dimensional (spatiotemporal) consistency of local neural activity (FOCA) is a local measurement method first proposed by Dong et al., 2015. It reflects both the temporal homogeneity of local regions and the spatial stability of spontaneous brain activity signals between adjacent time points (Dong et al., 2015). It has been increasingly used in neurologic diseases such as pediatric bipolar disorder (Gao et al., 2021; Guo et al., 2021), frontal lobe epilepsy (Dong et al., 2016), generalized tonic–clonic seizures (Ma et al., 2017), and schizophrenia (Chen et al., 2017). FOCA may provide additional information that can help in understanding brain function (Dong et al., 2015). A high FOCA value indicates low levels of temporal fluctuations and high regional stability. However, the use of this new method of integrating local temporal and spatial information in SVD has not been reported.

Although FOCA can provide a comprehensive view of the time series information of the brain, its application in understanding SVD remains unexplored. This study aimed to fill this research void by investigating the specific effects of SVD on various brain regions using FOCA. Furthermore, we sought to elucidate the relationship between these effects and the cognitive decline often associated with SVD. The findings of this study might contribute substantially to the understanding of the neurophysiologic mechanisms of SVD using the detailed temporal and spatial information provided by FOCA.

## 2 Materials and methods

### 2.1 Participants

All research procedures were approved by the hospital Ethics Committee (protocol number: Ethics (Research) No. 98, 2021), and all participants signed written informed consent. Fifty-three SVD patients presenting to our hospital between October 2021 and August 2023 were retrospectively included in this study. The inclusion criteria for patients with SVD were as follows: (1) diagnosis of SVD based on MRI imaging features according to the “Chinese Consensus on Diagnosis and Therapy of Cerebral Small Vessel Diseases 2021” (Hu et al., 2021); (2) no magnetic resonance imaging (MRI) contraindications such as claustrophobia; and (3) absence of notable structural brain abnormalities. The exclusion criteria for patients with SVD were as follows: (1) neurodegenerative diseases or mental disorders such as Alzheimer’s disease, Parkinson’s disease, or schizophrenia; (2) unrelated neurologic diseases such as epilepsy, stroke, intracranial hemorrhage, or brain tumors; (3) traumatic brain injury or metabolic encephalopathy; and (4) inability to complete the questionnaire due to auditory and visual impairment, speech impairment, or paralysis.

Forty-one healthy controls (HCs) comprised local community residents recruited through advertising. All HCs were required to complete a questionnaire to determine their information on general data and past medical history. In addition, all HCs were required to complete cognitive scale tests, including Mini-Mental State Examination (MMSE). The inclusion criteria for the HCs were no history of neurologic or psychiatric disorders, no cognitive disorders, and no abnormalities in conventional brain MRI images. Also, HCs with no cognitive impairment (MMSE score ≥ 25), stroke, or cerebrovascular disease were included. The detailed demographic information on SVD patients and HCs was provided in the results section.

### 2.2 MRI acquisition

All participants underwent conventional MRI, rs-fMRI, and structural MRI on a 3T MRI scanner (MAGNETOM VIDA, Siemens Healthcare, Erlangen, Germany) with a 64-channel head coil. The rs-fMRI images were acquired using gradient echo planar imaging sequence with the following parameters: repetition time (TR)/echo time (TE) = 1500/30 ms; flip angle = 70°; field of view (FOV) = 240 × 240 mm^2^; matrix = 94 × 94; thickness = 3 mm; gap = 0.75 mm; number of slices = 36; and voxel size = 2.5 × 2.5 × 3 mm^3^. The total scan time was 6 min 33 s, and a total of 255 images were acquired. Structural MRI scans were acquired using the magnetization prepared 2 rapid acquisition gradient echoes (MP2RAGE) sequence with the following parameters: TR/TE/inversion time (TI1)/TI2 = 5000/2.98/700/2500 ms; flip angle = 4°/5°; matrix = 256 × 256; FOV = 256 × 256 mm^2^; thickness = 1.0 mm; number of slices = 176; and voxel size = 1 × 1 × 1 mm^3^.

### 2.3 Data preprocessing

The rs-fMRI data were preprocessed using Statistical Parametric Mapping,^1^ as implemented in the Neuroscience Information Toolbox vision 1.3,^2^ (Dong et al., 2018). The rs-fMRI data were preprocessed as follows: the first 10 volumes were removed from the rs-fMRI data to ensure that the data is in a stable state; slice time correction; realignment; spatial normalization using individual structural MRI (normalizing to Montreal Neurological Institute space with 3 × 3 × 3 mm^3^); and smooth (full width at half maximum, FWHM = 8 mm). The participants with excessive head movement (translation > 2.5 mm or rotation > 2.5 mm) and mean framewise displacement (mFD) greater than 0.5 mm were excluded to avoid the impact of excessive head movement on the subsequent analysis.

### 2.4 FOCA analysis

The process for calculating the FOCA map for all participants using the NIT v1.3 software involved several steps (Dong et al., 2015). Before generating the FOCA maps, the software first removed various nuisance signals from the unsmoothed rs-fMRI data. These signals included 12 parameters related to head motion, average signals from white matter and cerebrospinal fluid, and any linear trends. Once these signals were removed, the software calculated the FOCA value for each voxel. Then, the FOCA value of each voxel was computed by integrating the temporal correlation among neighboring voxels (26 in total) and the spatial correlation between adjacent time points. After computing the FOCA values for all voxels throughout the brain, these values were normalized. This normalization involved dividing the FOCA value of each voxel by the average FOCA value across the entire brain. Following normalization, the mean FOCA maps were created and then smoothed using an 8-mm FWHM Gaussian kernel. Finally, the whole brain was segmented into 116 distinct regions using the Anatomical Automatic Labeling system, which served as masks for the analysis.

### 2.5 Total MRI burden of SVD

The SVD score was determined using a previously described 4-point scale with neuroimaging characteristics of SVD, including periventricular WMH or deep WMH, CMBs, LI, and perivascular spaces (PVS) (Staals et al., 2014). The WMH were defined as lesions appearing hyperintense on T2-weighted fluid-attenuated inversion recovery images and graded according to the Fazekas scale (Fazekas et al., 1987; Wardlaw et al., 2013). If the periventricular WMH Fazekas score was 3 or deep WMH Fazekas score was 2 or 3, 1 point was counted. The CMBs were identified and rated using the microbleed anatomical rating scale (Gregoire et al., 2009), and 1 score was counted if CMB was present. The LI was defined as a round or ovoid, subcortical, fluid-filled cavity between 3 and 15 mm in diameter under the cortex (Wardlaw et al., 2013), and 1 point was counted if CMB was present. The PVS were defined as small (<3 mm) structures of high signal on T2-weighted images and low signal on T1-weighted images located in the cerebrospinal fluid or basal ganglia centrum semiovale that followed the orientation of the perforating vessels and ran perpendicular to the brain surface (Doubal et al., 2010). The PVS grade was moderate to severe (2–4) with a score of 1 point. The SVD score ranged from 0 to 4. The images were scored independently by two experienced radiologists. If the results are inconsistent, the two radiologists decide by consensus.

### 2.6 Statistic analyses

Statistical analyses were performed using the SPM12. The two-sample *t* test was used to analyze FOCA differences between groups, using age, sex, education, head motion (mFD), and total intracranial volume as covariates. Pearson correlation analysis was used to analyze the association between brain regions with intergroup FOCA differences and total SVD scores. The criterion for statistical significance was *P* < 0.05, with a false discovery rate (FDR) used to correct for multiple comparisons (cluster size > 23).

## 3 Results

### 3.1 Demographic and clinical characteristics of the participants

A total of 53 patients with SVD and 41 HCs were recruited in this study. Among the patients with SVD, 7 with excessive head movement, 3 with kidney disease, and 1 with cerebral hemorrhage were excluded. In the control group, 3 HCs were excluded due to excessive head movement. Finally, 42 patients with SVD [19 male, 23 female; age (mean ± standard deviation), 68.524 ± 8.805 years], and 38 HCs [15 male, 23 female; age (mean ± standard deviation), 56.237 ± 4.210 years] remained in the final study. No differences were noted in sex, hyperlipidemia, smoking, and drinking. The demographic information and clinical characteristics are presented in Table 1.

**TABLE 1:** Demographic information of the healthy controls and patients with SVD.

	SVD (*n* = 42)	HCs (*n* = 38)	*P*-value
Age (year)	47–85 (68.524 ± 8.805)	49–67 (56.237 ± 4.210)	<0.05
Sex (male/female)	19/23	15/23	0.602
Years of education	0–12 (5.476 ± 3.238)	5–16 (10.145 ± 2.490)	<0.05
Vascular risk factors			
Hypertension	27 (64.286%)	6 (15.789%)	<0.05
Diabetes	14 (33.333%)	0	<0.05
Hyperlipidemia	7 (16.667%)	4 (10.526%)	0.964
Smoking	11 (26.190%)	8 (21.053%)	0.590
Drinking	12 (28.571%)	8 (21.053%)	0.438

Data were expressed as the range from min to max (mean ± SD) or number (percentage). *P*-value < 0.05 was considered to be statistically significant. HCs, healthy controls; SD, standard deviation; SVD, cerebral small vessel disease.

### 3.2 Analyzing FOCA values to differentiate SVD from HCs

**FIGURE 1:**
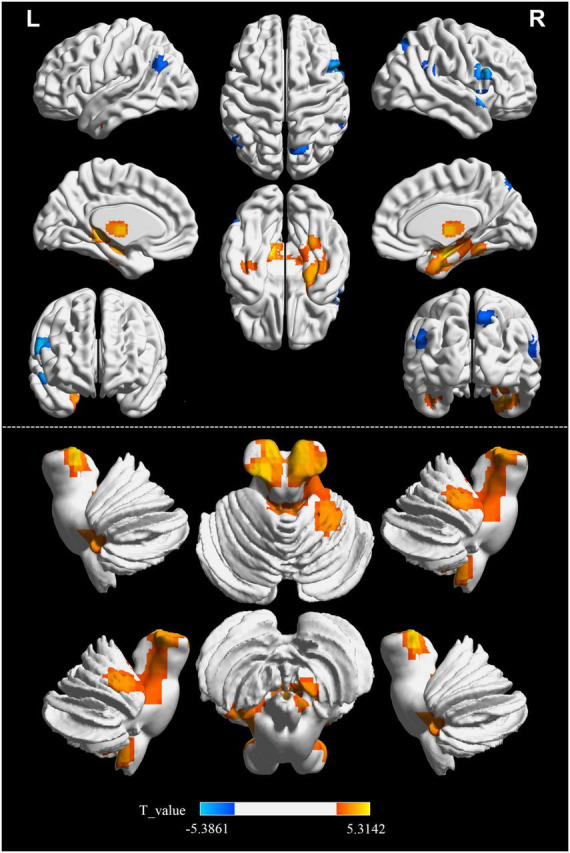
Differences in FOCA values between SVD and HCs groups. Cool color represents that the FOCA values was lower in the SVD group than in the HCs group (*P* < 0.05, FDR corrected, and cluster size > 23). FDR, false discovery rate; FOCA, four-dimensional (spatiotemporal) consistency of local neural activity; HCs, healthy controls; SVD, cerebral small vessel disease.

Significant differences in the FOCA map of SVD were demonstrated using the two-sample *t* test (*P* < 0.05, FDR corrected, and cluster size > 23). Compared with HCs, the decreased FOCA values of SVD were located in the right frontal_inf_oper, right temporal_pole_sup, right precuneus, right parietal_pup, right supramarginal, and left angular regions. The increased FOCA values of SVD were mainly observed in a few bilateral brain regions, including hippocampus, thalamus, cerebelum_9, and fusiform. The increased FOCA values were also observed in right parahippocampal, right cerebelum 4_5, left temporal_inf, left pallidum, left putamen, left cerebelum_10, and vermis_4_5 regions (Figure 1 and Table 2).

**TABLE 2:** Detailed information on significant differences in each brain region between SVD and HCs groups.

Region	MNI coordinates			Peak *t*_value	Cluster size
	*X*	*Y*	*Z*		
Hippocampu_L	−18	−33	0	4.87	347
Thalamus_L	−6	−12	3	4.46	
Thalamus_R	6	−12	3	4.34	
Hippocampus_R	36	−9	−18	4.61	407
Fusiform_R	30	−15	−27	4.14	
ParaHippocampal_R	24	−6	−33	4.1	
Cerebelum_4_5_R	18	−30	−27	3.42	
Temporal_Inf_L	−42	−6	−33	3.29	71
Fusiform_L	−39	−18	−27	3.2	
Putamen_L	−30	−6	−6	4.15	39
Pallidum_L	−15	0	0	4.25	
Vermis_4_5	−3	−48	−21	4.3	96
Cerebelum_9_R	9	−45	−54	3.87	29
Cerebelum_10_L	−24	−36	−39	3.85	38
Cerebelum_9_L	−18	−45	−42	3.33	
Temporal_Pole_Sup_R	60	15	−6	-5.18	61
SupraMarginal_R	54	−45	27	-3.82	25
Frontal_Inf_Oper_R	54	12	24	-5.38	79
Angular_L	−51	−66	27	-3.77	40
Parietal_Sup_R	18	−66	48	-3.98	47
Precuneus_R	12	−70	48	-3.27	

HCs, healthy controls; MNI, Montreal Neurological Institute; SVD, cerebral small vessel disease.

### 3.3 Correlation analysis between total SVD score and FOCA values

**FIGURE 2:**
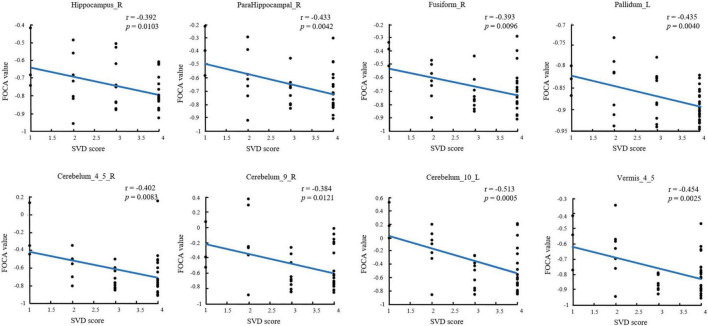
Substantial correlations between FOCA values in the brain regions and SVD score (*P* < 0.05, FDR corrected). FDR, false discovery rate; FOCA, four-dimensional (spatiotemporal) consistency of local neural activity.

The significant correlations between altered brain function value and SVD score are illustrated graphically in Figure 2 (*P* < 0.05, cluster-level FDR corrected). The SVD score had a negative correlation with the FOCA values of the right hippocampus (*r* = − 0.392, *P* = 0.0103), right parahippocampal (*r* = − 0.433, *P* = 0.0042), right fusiform (*r* = − 0.393, *P* = 0.0096), left pallidum (*r* = − 0.435, *P* = 0.0040), right cerebelum_4_5 (*r* = − 0.402, *P* = 0.0083), right cerebelum_9 (*r* = − 0.384, *P* = 0.0121), left cerebelum_10 (*r* = − 0.513, *P* = 0.0005), and Vermis_4_5 (*r* = − 0.454, *P* = 0.0025).

## 4 Discussion

This study was novel in investigating the spatiotemporal consistency of local brain spontaneous activity in patients with SVD based on the FOCA value of rs-fMRI. Considerable differences in FOCA values were noted between patients with SVD and HCs. Patients with SVD exhibited decreased FOCA values in certain right and left brain regions but increased FOCA values primarily in the bilateral hippocampus and other specified areas. The negative correlations between SVD scores and FOCA values in specific brain regions provided a preliminary understanding of the impact of SVD on brain function.

Previous studies have demonstrated that FOCA analyzes local spatiotemporal consistency, emphasizing local temporal homogeneity and regional stability of brain activity states (Dong et al., 2015, 2016). This study revealed that patients with SVD had reduced FOCA values in the right precuneus, parietal_sup, supramarginal, and left angular regions, which are parts of the default mode network (DMN). The DMN is a large-scale network consisting of a highly connected cortex combined with self-referential function, emotion, and episodic memory retrieval (Davey et al., 2016; Roberto et al., 2016; Smallwood et al., 2021). We observed reduced FOCA values in these brain regions, suggesting that DMN in patients with SVD may have abnormal spontaneous neural activity. Previous studies have also demonstrated that DMN is a commonly affected network in SVD (Li et al., 2023). This study observed reduced FOCA values in the right frontal_inf_oper and temporal_pole_sup regions. Previous studies have shown that patients with SVD have reduced functional connectivity in the frontal and temporal lobes (Sun et al., 2011; Gu et al., 2022; Yin et al., 2022). Vascular risk factors such as hypertension and diabetes affect the gray matter volume in the frontal and temporal lobes (Gold et al., 2005; Li et al., 2021; Zhang et al., 2022). In this study, SVD patients with hypertension or diabetes were not excluded. This may affect our results due to vascular risk factors. Therefore, we speculate that the decrease in FOCA values in the frontal and temporal lobes of SVD may be caused by multiple factors such as vascular risk factors and decreased connectivity. Our speculation will be confirmed in future work.

Moreover, this study reported increased FOCA values in the hippocampus, thalamus, pallidum, and putamen of patients with SVD, indicating enhanced coordination and stability of neural activity within these regions. Similar results were obtained in studies of other SVD subtypes (Feng et al., 2021; Hu et al., 2022). Several studies have demonstrated that the hippocampus/parahippocampal gyrus is essential for the brain’s long-term episodic memory, semantic memory, spatial cognition, learning, emotion, and more (Danjo et al., 2018; Loprinzi, 2019; Maurer and Nadel, 2021; Peng and Burwell, 2021). Meanwhile, the putamen, pallidum, and thalamus are the main components of the basal ganglia located in the deep brain region. The basal ganglia are thought to be crucial in learning and memory. They are associated with executive decision-making and reward, cognition, emotion, and function (Graybiel, 2000; Florio et al., 2018). Further, we demonstrated that the FOCA values of the hippocampus, parahippocampal and pallidum were negatively correlated with the SVD score. A recent study also confirmed that the SVD score was significantly negatively correlated with the overall cognitive function and attention (Hosoya et al., 2023). Linking FOCA values to cognitive functions through SVD scores suggests a hypothesis: alterations in FOCA values might serve a compensatory role. Enhancing the spatiotemporal consistency in brain regions with decreased FOCA values could potentially compensate for neuronal damage and cognitive deficits in these areas, thereby helping to maintain normal daily functions. Further research is needed to validate this hypothesis, ideally through studies that integrate more comprehensive assessments of brain connectivity and cognitive functions to better understand the underlying mechanisms.

Traditionally, the cerebellum has been considered the brain region associated with movement; however, a growing body of research has demonstrated its involvement in more complex functions, including emotional regulation, social processing, and cognitive function. The anterior cerebellar lobe has extensive connections with sensorimotor function, whereas the posterior cerebellar lobe (crus I and VIIb) is involved in cognitive function. The vermis is known to have extensive connections with the limbic structures of the brain, thus demonstrating the involvement of the cerebellum in emotion and emotional behavior (Leggio and Olivito, 2018; Schmahmann, 2019; Pierce and Péron, 2020). This study demonstrated an increase in FOCA values in the vermis. Further, we demonstrated that the FOCA values of cerebellum were negatively correlated with the SVD score. This finding was consistent with previous studies where Ding et al. suggested an increase in ALFF in the cerebellum (Ding et al., 2015), which might be an attempt to recruit more neurons to compensate for cognitive impairment. In addition, the present study demonstrated an increase in FOCA values in the anterior cerebellar region, which was rarely reported in previous studies. This might be because most previous studies focused on the SVD subtype. Another aspect might be the additional information derived using the FOCA method. This might provide new insights into the pathophysiologic mechanisms in patients with SVD. Moreover, several studies have demonstrated that the basal ganglia are closely linked to the cerebral cortex and cerebellum to form a complete network, namely the basal ganglia–cerebellar–cortical network, which is involved in various motor and cognitive functions. However, this is considered to be a complete system that allows us to understand the function of these regions using a completely unique approach (Bostan and Strick, 2018; Milardi et al., 2019; Pierce and Péron, 2020). Future studies should investigate the changes in the loop in patients with SVD.

## 5 Limitations

This study has a few limitations. Firstly, an age mismatch was seen between patients with SVD and HCs. Therefore, in the statistical analysis, age was removed as a covariate to reduce its impact on the reliability of the results. Secondly, the SVD patients in this study lacks professional scale to evaluate the cognitive function, such as MMSE, which will be further studied in future work.

## 6 Conclusion

Our results suggest that there is a wide range of spontaneous brain activity abnormalities in patients with SVD, which may be related to cognitive impairment in patients with SVD, and the FOCA method may provide a powerful tool for further understanding the underlying neurophysiological mechanisms of SVD.

## Data availability statement

The raw data supporting the conclusions of this article will be made available by the authors, without undue reservation.

## Ethics statement

The studies involving humans were approved by the Ethics Committee of Zigong First People’s Hospital. The studies were conducted in accordance with the local legislation and institutional requirements. The participants provided their written informed consent to participate in this study.

## Author contributions

JY: Methodology, Writing – original draft. RX: Methodology, Writing – original draft. YL: Data curation, Writing – original draft. CH: Investigation, Writing – original draft. LH: Validation, Writing – original draft. XX: Project administration, Writing – review and editing. MC: Writing – review and editing. JZ: Project administration, Writing – review and editing.

## Footnotes

1
https://www.fil.ion.ucl.ac.uk/spm/software/spm12/

2
http://www.neuro.uestc.edu.cn/NIT.html
